# Understanding the complexity of sepsis mortality prediction via rule discovery and analysis: a pilot study

**DOI:** 10.1186/s12911-021-01690-9

**Published:** 2021-11-28

**Authors:** Ying Wu, Shuai Huang, Xiangyu Chang

**Affiliations:** 1 Center for Intelligent Decision-Making and Machine Learning, School of Management, Xi’an Jiaotong University, No.28, Xianning West Road, Xi’an, 710049 People’s Republic of China; 2 Department of Industrial and Systems Engineering, University of Washington, Seattle, USA

**Keywords:** Sepsis, RuleFit, Mortality prediction

## Abstract

**Background:**

Sepsis, defined as life-threatening organ dysfunction caused by a dysregulated host response to infection, has become one of the major causes of death in Intensive Care Units (ICUs). The heterogeneity and complexity of this syndrome lead to the absence of golden standards for its diagnosis, treatment, and prognosis. The early prediction of in-hospital mortality for sepsis patients is not only meaningful to medical decision making, but more importantly, relates to the well-being of patients.

**Methods:**

In this paper, a rule discovery and analysis (rule-based) method is used to predict the in-hospital death events of 2021 ICU patients diagnosed with sepsis using the MIMIC-III database. The method mainly includes two phases: rule discovery phase and rule analysis phase. In the rule discovery phase, the RuleFit method is employed to mine multiple hidden rules which are capable to predict individual in-hospital death events. In the rule analysis phase, survival analysis and decomposition analysis are carried out to test and justify the risk prediction ability of these rules. Then by leveraging a subset of these rules, we establish a prediction model that is both more accurate at the in-hospital death prediction task and more interpretable than most comparable methods.

**Results:**

In our experiment, RuleFit generates 77 risk prediction rules, and the average area under the curve (AUC) of the prediction model based on 62 of these rules reaches 0.781 ($\pm 0.018$) which is comparable to or even better than the AUC of existing methods (i.e., commonly used medical scoring system and benchmark machine learning models). External validation of the prediction power of these 62 rules on another 1468 sepsis patients not included in MIMIC-III in ICU provides further supporting evidence for the superiority of the rule-based method. In addition, we discuss and explain in detail the rules with better risk prediction ability. Glasgow Coma Scale (GCS), serum potassium, and serum bilirubin are found to be the most important risk factors for predicting patient death.

**Conclusion:**

Our study demonstrates that, with the rule-based method, we could not only make accurate prediction on in-hospital death events of sepsis patients, but also reveal the complex relationship between sepsis-related risk factors through the rules themselves, so as to improve our understanding of the complexity of sepsis as well as its population.

**Supplementary Information:**

The online version contains supplementary material available at 10.1186/s12911-021-01690-9.

## Background

### Introduction

Sepsis is defined as life-threatening organ dysfunction caused by a dysregulated host response to infection, according to the Sepsis-3 definitions [1], and is one of the leading causes of death in ICUs in the U.S. Severe sepsis as well as septic shock can be viewed as the more severe stages of sepsis and are both associated with a dramatic increase in mortality. It is reported in 2001 that each year in the U.S., nearly 750,000 patients are identified with sepsis or sepsis-related disease and over half of them are admitted to ICU. Among these ICU cases, 20-30% died in the hospital [2]. Moreover, sepsis and related disease have posed a great challenge to the already strained finances of hospitals: in 2011, about 5.2% ($20 billion) of total U.S. hospital costs were related to sepsis [3], which ranked top in the four most costly conditions in the hospital [4]. More importantly, the incidence of sepsis increased by 13% annually between 2004–2009, based on four national data sources [5].

Being a syndrome, rather than a specific illness, sepsis is still uncertain with respect to its pathology. It is recognized to be associated with many abnormalities in the body systems and functions like cardiovascular, neuronal, metabolic systems and coagulation, along with early activation of both pro- and anti-inflammatory responses [1]. In addition, the complexity of sepsis ascends as sepsis-affected individuals show diverse manifestations in aspects of age, source of infection, underlying comorbidities, and concurrent injuries. The heterogeneity and complexity of sepsis lead to the absence of gold standards for its diagnosis, treatment, and prognosis [6].

Prediction of clinical outcomes of patients with sepsis in ICUs is a vital research task. Length of hospital stay or ICU stay [7, 8], in-hospital mortality [9, 10], and readmission [8, 11] are often considered as the outcomes of interest. The early prediction of these outcomes are not only associated with improving the well-being of patients, but also relates to the evaluation of quality-of-care of health centers, the performance of healthcare practitioners, and the effectiveness of clinical decision making [12, 13]. Improved predictions help to stratify ICU patients into different risk categories and contribute to personalized care and treatment. Though many efforts have been made in this direction [14–17], improved mortality prediction remains a challenge due to the complexity and heterogeneity of sepsis.

In this study, we apply a rule-based method on a sepsis data set to predict the in-hospital death events of the sepsis population. We use RuleFit [18] to generate a set of rules and based on part of these rules, we build our prediction model of in-hospital mortality risk. Both the internal validation and external validation demonstrate that the rules we discover are not only capable of mortality prediction for sepsis patients but are also informative in helping us understand the complexity of sepsis and its population. For better report of the entire study, we provide a TRIPOD Checklist with added text excerpts or relevant remarks in Additional file 2.

### Related work

Roughly speaking, existing research on prediction of sepsis mortality could be categorized into two main approaches: regression-based scoring systems and machine-learning-based prediction models. A number of scoring systems have been developed to assist the assessment of disease severity as well as risk of mortality of critically ill patients, among which APACHE II [19], SAPS II [20], SOFA [21], and MODS [22] are the most frequently used.

Among the scores mentioned above, some are general risk-prognosis systems aimed at assessing the overall health condition of patients, such as APACHE II and SAPS II, whereas others like SOFA, MODS, and several newly developed prediction models, say, The New York Sepsis Severity Score [23], are developed specifically for patients with sepsis or related diseases. In general, the ability of general scoring systems to predict sepsis patient prognostic outcomes is not always reliable, consistent, and sufficiently accurate compared with that of the specific systems, which has been acknowledged in many studies [14, 15, 24]. Moreover, a big concern with all these scoring systems is that they are mainly derived via regression models that rely on quite strong assumptions such as the linearity of model, additive effects (rather than interactions) of the risk factors on the outcome and identical normal physiologic values at baseline for all patients [25]. Thus, these models are mainly used to stratify patients into different risk categories and make predictions on the average population [11, 26].

For the purpose of better prediction, many machine learning models have been applied on a large number of potential risk factors of sepsis mortality in determining the outcomes, such as decision tree [10], random forest [17], neural network [10], naïve Bayes [16], gradient boosting [27], and ensemble learning method [9]. These models are shown to outperform traditional scoring systems in terms of prediction accuracy and reliability. Nevertheless, compared with the traditional scoring systems, machine learning models are usually black boxes the results of which are not easy to interpret [28]. As interpretability has become a crucial concern for healthcare applications of machine learning [28, 29], it is imperative to elevate our understanding of the disease while we strive for better prediction performance, in order to establish a prediction model more acceptable, feasible, and pragmatic to healthcare practitioners. Therefore, we aim to predict sepsis in-hospital mortality with comparable accuracy to the above baseline models and at the same time, achieve better interpretation.

**Table 1:** Baseline statistics of the sepsis subjects

	In-hospital death	Survivors
	N= 675	N= 1346
	Mean (SD)	Mean (SD)
**Demographics**		
Age	66 (17.8)	69.7 (15.5)
Gender n(%)		
Male	370 (54.8%)	716 (53.2%)
Female	305 (45.2%)	630 (46.8%)
Ethnicity n(%)		
Asian	22 (3.2%)	51 (3.8%)
Black	46 (6.8%)	115 (8.5%)
Hispanic/Latino	13 (1.9%)	44 (3.3%)
White	473 (70.1%)	973 (72.3%)
Others	121 (17.9%)	163 (12.1%)
**Admission info (day)**		
Length of hospital stay	8.0 (10.3)	11.9 (10.7)
Length of ICU stay	6.6 (8.3)	7.6 (9.4)
**Measurements**		
Glasgow Coma scale	11 (3)	9(3)
Temperature	37 (1.6)	36.4 (1.8)
Mean arterial pressure	68.8 (46.8)	64.9 (56.8)
Arterial pH	7.3 (0.1)	7.2 (0.2)
Heart rate	98.4 (33.1)	101.7 (43.3)
Respiratory rate	20.3 (16)	17.5 (19.5)
Sodium	139.1 (6.6)	137.7 (7.3)
Serum potassium	4 (0.7)	4.4 (0.9)
Creatinine	1.7 (1.5)	2.3 (1.7)
Hematocrit	30.8 (5.8)	30.6 (6.7)
White blood cell count	15.6 (10.7)	17.2 (20.8)
Albumin	2.6 (0.4)	2.5 (0.5)
Bilirubin	1.8 (3)	3.5 (6)
Platelets	216.2 (139.8)	197 (152.8)

## Methods

### Data

We use data from the Medical Information Mart for Intensive Care database (MIMIC-III), a freely accessible critical care database [30]. This database contains information related to patients admitted to ICU at a large tertiary care hospital between 2001 and 2012 in the U.S. There are records of 46,520 patients and 58,976 admissions in the database, including information like patient demographics, vital sign measurements, laboratory (lab) test results, procedures, medications, caregiver notes, imaging reports and others.

The MIMIC-III database has been used in a broad range of research topics such as the impact of certain factors on patients’ clinical outcomes [31], the development and validation of ICU severity scoring systems and the comparison of different systems [13, 32]. Machine learning, deep learning, reinforcement learning as well as natural language processing applications on records of various forms [33–36]. As to sepsis, researchers have been making great efforts to improve the early detection, diagnosis, and treatment of this syndrome and there have been a number of relevant studies using MIMIC-III [13, 35, 37–39]. In order to find important risk factors and informative rules for the prediction of in-hospital death of sepsis patients, we extract the sepsis data set from MIMIC-III for the purpose of our study. The data set involves a subset of MIMIC-III patients who are diagnosed with sepsis, severe sepsis, or septic shock based on the ICD-9 [40] code suggested in [1]. The patient inclusion criteria of our cohort are: (i) age $\ge$ 16 ; (ii) only has one hospital admission record and one ICU admission record; (iii) with ICD-9 code of 99591 for sepsis, 99592 for severe sepsis, or 78552 for septic shock. We manually collate the data into 2021 observations (patients) with 19 predictors, 14 of which are physiological measurements or lab test results shown in Table 1. Missing values are imputed with the k-nearest neighbors imputation method implemented with R package *DmWR2* (the number of nearest neighbours is set to be 5). The worst value of each predictor within 24hr of ICU admission (for definition and computation, refer to [41] and Additional file 3) is computed and used to fit the RuleFit model. The outcome in our study is defined to be any death events of patients from the second day of ICU admission to hospital discharge. Table 1 shows the descriptive statistics of the measurements used in our sepsis data set. Note that the selection of predictors as well as the way we extract the features from the continuous records are for illustration and example only. More informative predictors such as comorbid conditions, source of infection can be included if available. Apart from the worst values, other common feature extraction standards like initial values, quantiles of predictors, or a mix of these standards can be employed based on specific scenarios.

**Fig. 1:**
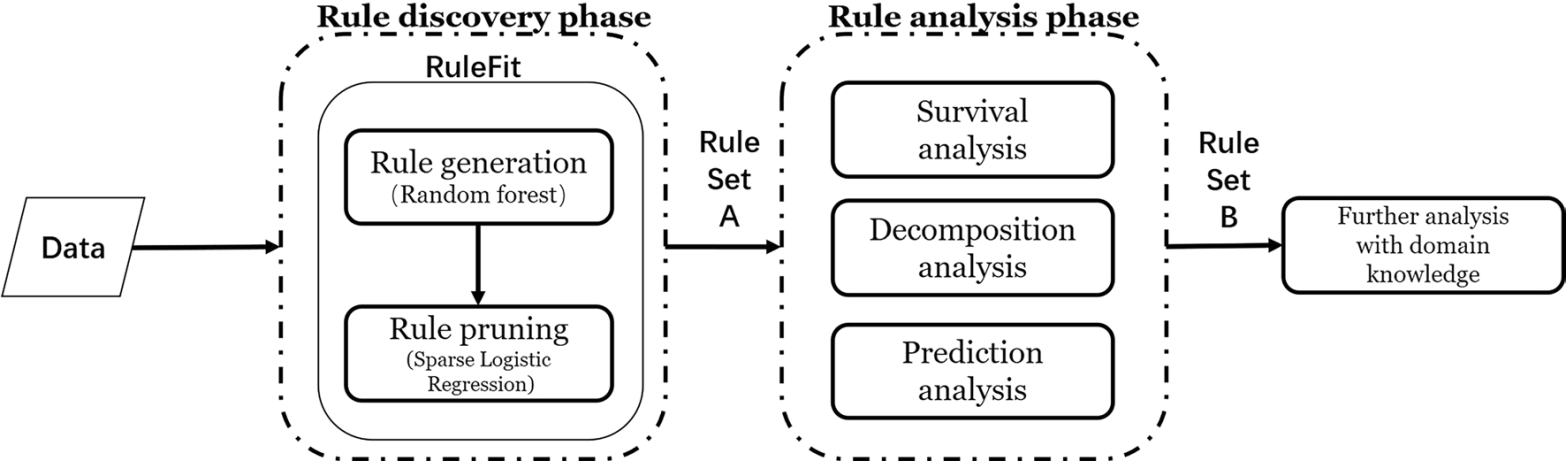
Workflow of the rule-based method

### Overview of the rule-based method

**Fig. 2:**
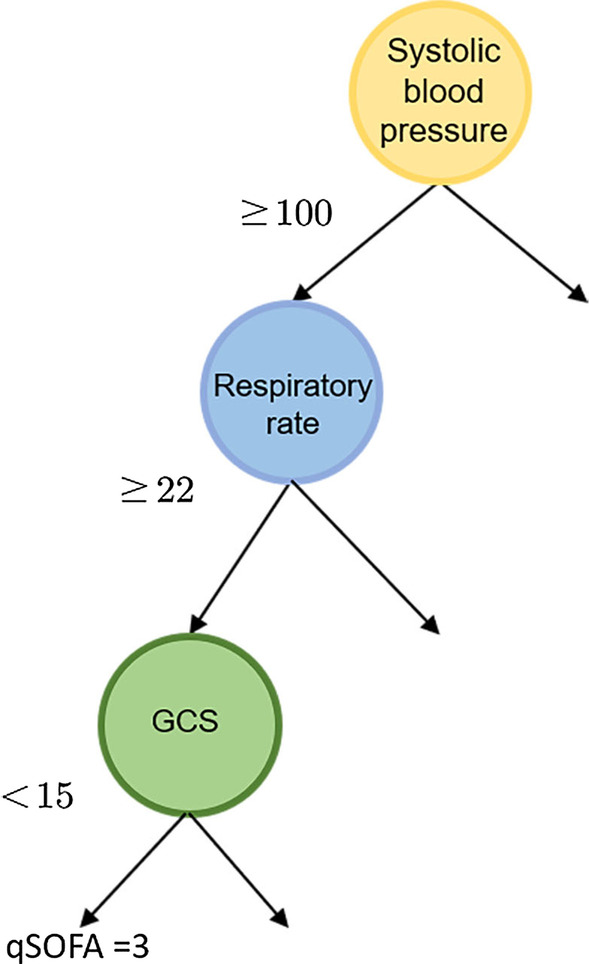
Rule of qSOFA computation. The path from the root to the leftmost leaf node of the tree represents the rule of scoring three points of qSOFA

We use a rule-based method to predict the in-hospital death events of sepsis patients in our study. Figure 1 shows the workflow of the method. In the rule discovery phase, RuleFit [18] is used to discovery potential and possible rules from the data and a rule set A is produced. Then in the rule analysis phase, we analyze rules in the rule set A and obtain a more refined rule set B, the rules of which are more discriminative and informative than those of rule set A on the task of risk prediction. Further detailed analysis could be made on rule set B with expert knowledge. Now we give a detailed description of our workflow.

As its name implies, the rule-based method builds on multiples rules. A rule is defined as a logic IF-THEN statement: **IF** condition **THEN** conclusion [42]. Take the computation of qSOFA (a quick scoring prompt used to identify patients with suspected infection who are at greater risk of clinical outcomes outside the ICU) [1] as an example: If the systolic blood pressure, respiratory rate, and GCS of a patient are not greater than 100 mmHg, not less than 22 bpm, and less than 15 respectively, then the qSOFA score of the patient is three points. Also, we can represent this statement with a tree form as illustrated in Fig. 2 where the path from the root to the leftmost leaf node is equivalent to the statement.

Rules are a natural way to represent information and knowledge. Rules are easily understood, repeated, and modified by human experts [42], which we suppose is of great significance for healthcare studies and practices, especially on complex disease like sepsis: Rules may help us understand the complexity of sepsis by revealing different risk patterns of patients (cut-off values of risk factors and unknown interactions between these risk factors). By making the prediction more interpretable, rules enhance our understanding of a patient’s adverse outcome. In summary, rules help us understand sepsis and the population in a more interpretable way.

### Rule discovery via RuleFit

In our study, we use the RuleFit method to mine the hidden rules which are indicative of patient’s in-hospital death. RuleFit has many advantages over other rule generation methods (e.g., decision tree): (i) RuleFit is able to discover a large number of rules from high-dimensional data and in a computationally efficient way; (ii) RuleFit can automatically remove redundant and irrelevant rules from a pool of rules; (iii) RuleFit offers multiple methods to make its result more interpretable, such as rule importance, input variable importance, etc. These merits cater to our research expectation: we aim to discover high-quality risk-predictive rules from a quantity of potential rules revealing the complexity of sepsis mortality prediction.

In Rulefit, rule is formulated as a mapping: $r(\mathbf {x}) = \prod _{j=1}^{p} \mathbbm {1}_{x_j \in s_j}$, where $\mathbf {x}=(x_1,\dots ,x_p)^T \in \mathbb {R}^p$ denotes the *p*-dimensional feature (input variables) and $x_j$ denotes the *j*-th element of $\mathbf {x}$. $s_{j}$ is a specified subset of all possible values of $x_j$ and $\mathbbm {1}$ is the indicator function. Rule $r(\mathbf {x})$ maps an input $\mathbf {x}$ into $\{ 0,1 \}$ where 1 means the rule is satisfied and 0 otherwise. Given an input case $\mathbf {x}$ (without missing elements), we are able to determine whether a given rule is satisfied or not. For instance, in the example of Fig 2, we could define $s_1 = \{x \in N| x\le 100\}$, $s_2 = \{x \in N| x \ge 22\}$, $s_3 = \{x \in N| x\le 15\}$ (*N* represents the natural numbers) to be the condition of the rule. Suppose the systolic blood pressure, respiratory rate and GCS of a patient are 80 mmHg, 40 bpm, and 10 respectively, i.e., $\mathbf {x} = (80, 40, 10)^T$, then we obtain $r(\mathbf {x}) = 1$, which means the rule is satisfied and the qSOFA of this patient is three points.

Building on random forest [43] and LASSO regression [44], the RuleFit method [18] first derives a large number of rule ensembles from random forest in the rule generation step and then selects a subset of rules from the rule ensembles to form a more refined rule set in the rule pruning step. We then show in detail how RuleFit works.

#### Rule generation

In the rule generation step, random forest is used to discover possible rules from the data set. Random forest is an ensemble learning method for prediction tasks which can deal with high-dimensional data: it first bootstraps the original data set and generates multiple new data sets, each of which represents a relatively homogeneous sub-population of the original data set. Then on each data set, a decision tree is established by a subset of randomly selected risk factors and each tree could be viewed as a set of rules characterizing a sub-population, as shown in the example of Fig. 2. In this way, random forest is able to capture traits of the whole population and search for potential rules over all risk factors. As the generation mechanism for random forest trees is exhaustive, the number of rules generated goes exponentially with the number of risk factors. Besides, trees in random forest are constructed in a random way. For these reasons, many rules employed in the trees may be redundant and irrelevant and need to be removed in the subsequent rule pruning step.

#### Rule pruning

The rule pruning step could be viewed as a rule selection mechanism. Due to possible over-fitting of random forest that might derive a large number of unnecessary rules, LASSO regression is integrated in RuleFit as a way to select the minimum set of risk-predictive rules by using all rules generated in the previous step as predictors. As LASSO regression is an effective sparse learning approach to select the most critical variables from a large number of candidate variables, it could also be used to select a subset of high-quality of rules from a pool of candidate rules.

Since the outcome in our study is in-hospital death events, a binary outcome ($y \in \{-1,1\}$), we use sparse logistic regression (an extension of the original LASSO regression) [45] in our model. Suppose random forest generates a rule set with *q* rules which are denoted by $\mathbf {r} = [r_1,r_2,\dots ,r_q]^T \in \mathbb {R}^q$. Then the conditional probability of *y* given the risk factors of a patient $\mathbf {x} \in \mathbb {R}^p$ is:

1 $$\begin{aligned} \mathrm {p} (y|\mathbf {x}) = \frac{1}{1+\exp (-y(\mathbf {w}^T\mathbf {r}(\mathbf {x})+b))}, \end{aligned}$$

where $\mathbf {w} \in \mathbb {R}^q$ is the weight vector for the rules, and *b* is the intercept. Suppose we have *N* observations $\{ \mathbf {x}_i, y_i\}^{N}_{1=1}$, then the average logistic loss (also called the negative log likelihood function) is defined as:

2 $$\begin{aligned} f(\mathbf {w},b) = -\frac{1}{N} \mathrm {log}\prod ^{N}_{i=1} \mathrm {p}(y_i|\mathbf {x}_i) \end{aligned}$$

By adding $\ell _1$-norm penalty to $\mathbf {w}$ ( $\ell _1$-norm is defined as the sum of the absolute values of all elements in $\mathbf {w}$), we obtain the $\ell _1$-regularized logistic regression problem:

3 $$\begin{aligned} \mathop {\mathrm {min}}_{\mathbf {w},b} \frac{1}{N}\Sigma ^{N}_{i=1} \mathrm {log} (1+\exp (-y_i(\mathbf {w}^T\mathbf {r}(\mathbf {x}_i)+b))) + \lambda ||\mathbf {w}||_1. \end{aligned}$$

In problem (3), the first term is the loss term used to measure the model fit and the second term is the penalty term used to measure the complexity of the model where $\lambda > 0$ is the regularization parameter controlling the balance between model complexity and model fit: a larger $\lambda$ makes a more sparse $\mathbf {w}$ and thus fewer rules are selected. $\lambda$ needs tuning in order to avoid over-fitting as well as enhance sparsity. In this way, the most risk-predictive subset of rules would be selected. A number of efficient algorithms could be used to solve problem (3), such as subgradient-based algorithms like proximal gradient algorithms [45], etc.

In conclusion, as an integration of random forest and LASSO, RuleFit is a computationally efficient method to generate a number of predictive rules from high-dimensional data. It works by discovering rules with random forest and pruning rules with LASSO regression. More theoretical details on RuleFit can be found in [18]. RuleFit could be easily implemented with R package *pre* [46] which also provides automated cross-validation procedures to tune parameters in the model, such as the maximum depth of tree, average number of terminal nodes, and the penalty parameter $\lambda$.

### Rule analysis

The RuleFit method integrates random forest and LASSO regression [44] to generate a quite refined rule set which is predictive of the outcome of interest. However, due to the random tree generation algorithm and fake correlations between variables, RuleFit does not guarantee all rules in the refined rule set are informative and discriminative, even with the utility of LASSO. Therefore, the rules discovered by RuleFit are only possible rules that need to be further analyzed in terms of how well they perform in distinguishing patients of different death risk. In the rule analysis phase, we analyze the rule set discovered by RuleFit in three aspects: firstly, survival analysis to see if each of the rules is able to significantly distinguish the higher risk group of patients from the lower risk group. Secondly, decomposition analysis to investigate the role of each risk factor plays in each rule. Thirdly, prediction analysis to test the overall prediction power of the rules which are discriminative and informative.

#### Survival analysis

As each rule in the rule set discovered by RuleFit differentiates two groups of population (the higher risk group and the lower risk group), we can use Kaplan-Meier (KM) survival analysis and log rank test [47] to evaluate the discriminative power of each rule in distinguishing the higher risk group from the lower risk group. In the medical area, KM survival analysis is often used to estimate the survival function of certain patients and reflect their survival fraction over time. Log rank test is used to compare the survival distributions of two samples and test if the difference of the two samples is significant.

**Fig. 3:**
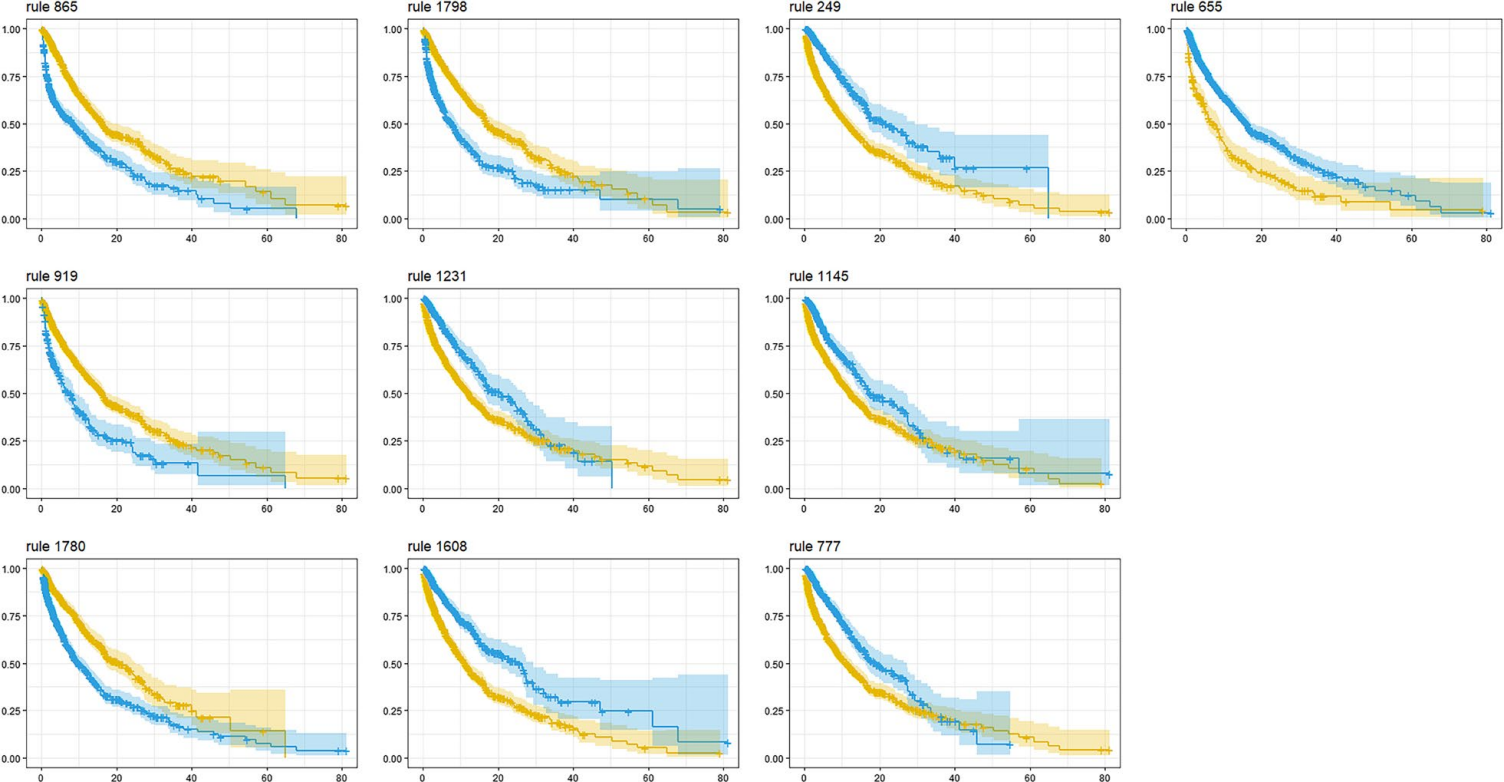
Kaplan–Meier survival curves (with 95% confidence interval) of the two groups defined by each rule: one endorses the rule (blue curve) and one does not (yellow curve). For each subplot, the horizontal axis represents time (day) and the vertical axis survival probability

In our study, if the difference of in-hospital mortality of different risk groups identified by each rule is significant enough, we may say the two groups identified by the rule are separated, thus the rule has the ability to identify different risk groups. The KM curves corresponding to a rule together with the *p* value given by the log rank test are able to tell how the two groups identified by the rule are separated and hence how discriminative the rule is. For illustration, the KM curves of a rule (e.g., rule 865) in Fig. 3 show that the group not endorsing the rule (yellow curve) generally has lower risk of mortality (higher survival fraction) than that of the group endorsing the rule (blue curve), and this gap of mortality significantly differs, which could be verified by a log rank test *p* value of 0 ($<0.001$) displayed in Table 2.

#### Decomposition analysis

A rule is usually composed of multiple risk factors, each of which may contribute to the predictive power of the complete rule. These risk factors in combination produce a synergistic effect where the whole is greater than the sum of the parts. Decomposition analysis can be done to evaluate the role of each risk factor in a given rule. In detail, we can decompose each rule into several parts where each part contains a risk factor (and the cut-off value with it), then we remove each risk factor in the rule respectively and obtain multiple revised rules. Survival analysis mentioned above could be applied to the revised rules, and by comparing the log rank test *p* value of each revised rule with that of the complete rule, we are able to know how important each risk factor is to the complete rule. If the complete rule shows better discriminative power than any revised rule (the *p* value for the complete rule is less than any *p* value for the revised rule), then it may suggest that each risk factor in the rule is necessary and that they together have a synergistic effect on defining different risk groups. Take rule 865 in Table 2 for instance: rule 865 could be decomposed into two parts, namely pH.art $\le$ 7.2 and Age > 46. In both cases, the *p* value for the rule when a risk factor is removed (1.29E-09 and 3.29E-14) is larger than the *p* value for the complete rule (0), indicating that both pH.art and age play a role in the discriminative power of the complete rule. In other words, any removal of risk factors from the complete rule will reduce the ability of rule 865 to distinguish the high risk group from the low risk group.

#### Prediction analysis

In this phase, we can use rules that show significant discriminative power in both the survival analysis and the decomposition analysis to build the in-hospital death prediction model for sepsis patients. In this way, the overall prediction power of these rules can be investigated and compared. In our study, we simply check the endorsement of patients on each rule and train a logistic regression model with multiple binary variables indicating whether the rule is satisfied for each patient. We clarify that the use of logistic regression model here is only an easy example to show how we could use the rules to predict in-hospital mortality. More complex and accurate prediction methods could also be used for this end. As rules incorporated in the regression model are already tested for their significant discriminative power in the previous two analysis, we assume the details of the regression model are trivial and thus do not report them.

Actually, the prediction analysis enables us to compare the prediction performance of the rule-based method with other common baseline methods (e.g., random forest) on the sepsis data. To do this, we can first tune the parameters with a 10-fold cross validation and obtain the optimal parameters for each baseline model as well as the rule-based model. Next, we split the data randomly into a 70% training set and 30% test set. Each model is trained with the training data and the optimal parameters, while performance metric like AUC is calculated merely on the test set. To avoid the randomness of the experiment, we can repeat this 70/30 split procedure 100 times for each model and report the mean AUCs. Note that in this process, the baseline models are fed with identical covariates. Furthermore, we could also examine the significance of difference in mean AUCs between these baseline methods and the rule-based method with the Delong test [48].

## Results

### Identification of risk-predictive rules on the sepsis patients

We apply RuleFit on the sepsis data to derive a set of risk-predictive rules as well as to predict the in-hospital mortality of this sepsis population. The RuleFit method does not need an explicit standardization of data since it utilizes random forest, a method which is able to deal with data of different scales. We manually impute the missing data by *K* Nearest Neighbors [49] with the number of neighbors set to be five. The binary individual outcome to be predicted is whether the patient will die in the hospital.

In our experiment, we set the maximum depth of tree to be three to avoid the occurrence of complex rule ensembles. We then tune the parameters, i.e., the number of trees generated by random forest and the degree of penalty $\lambda$, using the automated 10-fold cross validation procedure in RuleFit. The final optimal values of these parameters are determined by the corresponding misclassification rate. Our experiments show that the optimal number of trees is 375 and we derived the final rule set including 77 rules, the $\lambda$ of which is within one standard error of the minimum cross-validated error.

**Table 2:** Top 10 rules generated by RuleFit on the sepsis population and filtered by a simple mechanism

Rules	*P* value (log rank)
**865**(support=26%,direction=increasing)	0
pH.art $\le 7.2$	1.29E−09
Age $> 46$	3.29E−14
**919** (support=13%, direction=increasing)	3.77E−15
Potassium.serum $> 4.1$	6.32E−07
Platelets $\le 136.7$	1.92E−11
**1780**(support=46%, direction=increasing)	0
Creatine $>1.2$	0.000421
Potassium.serum $>3.3$	7.66E−15
**1798** (support=25%, direction=increasing)	0
Potassium.serum $>4.25$	2.46E−10
MAP $\le 59$	2.10E−11
**1231** (support=39%, direction=decreasing)	4.34E−11
GCS $\ge 9$	3.58E−09
Heart rate $\le 129$	2.75E−08
Creatine $\le 1.7$	2.47E−10
**1608** (support=39%, direction=decreasing)	1.11E−16
GCS $> 5$	1.57E−13
Bilirubin $\le 1.15$	8.80E−14
Age $\le 81$	2.19E−07
**249** (support=39%, direction=decreasing)	0
pH.art $> 7.2$	7.66E−15
creatine $\le 1.2$	3.29E−14
**1145**(support=41%, direction=decreasing)	1.44E−09
GCS $> 9$	0.004001
Bilirubin $\le 20$	9.17E−09
heart rate $\le 133$	3.13E−07
**777**(support=48%, direction=decreasing)	8.22E−15
GCS $\ge 8$	1.26E−13
Potassium.serum $\le 4.1$	4.10E−09
Albumin $> 1.9$	3.51E−13
**655**(support=89%, direction=decreasing)	1.12E−14
GCS $> 5$	1.92E−07
Bilirubin $\le 7.5$	6.71E−08

The first column shows the rule

The second column gives the *p* value of log rank test of each (revised) rule

Due to possible presence of rules that are not informative and discriminative, we further filter the 77 rules discovered by RuleFit with survival analysis and decomposition analysis procedures to obtain the rules that are relatively more convincing and reliable. Details of the filter criteria can be found in Additional file 3. After the filter process, 62 rules are retained, based on which we train our mortality prediction model. For conciseness, we only show the top 10 rules in Table 2 and discuss them in detail in the discussion section. The components, support, and direction of each rule are also shown in Table 2 where the support of a rule means the proportion of subjects endorsing the rule, and the direction of a rule means the direction of change in death risk if a subject endorses the rule. We also list the remaining 52 rules as well as the 15 discarded rules in Additional file 1.

### Analysis of the identified rules

For each rule discovered by RuleFit, we perform the KM analysis on the whole population and test the difference of mortality between the group endorsing the rule and the group not endorsing the rule through log rank test. The KM curves of the top 10 rules shown in Fig. 3 and the result of log rank test (*p* values) listed in Table 2 demonstrate that all the 10 rules have discriminative power to identify patients with different levels of mortality risk as the KM curves of each rule are quite separate and the mortality risk gaps are significant (*p* values $< 0.001$). Besides, the decomposition analysis of all 62 rules is also performed and the results for the top 10 are also listed in Table 2. It is apparent that each risk factor plays an important role in the complete rule since the *p* value of each rule with any risk factor being removed becomes not as significant (*p* value becomes larger).

### Assessment of the prediction performance of the rule-based method on sepsis population

**Fig. 4:**
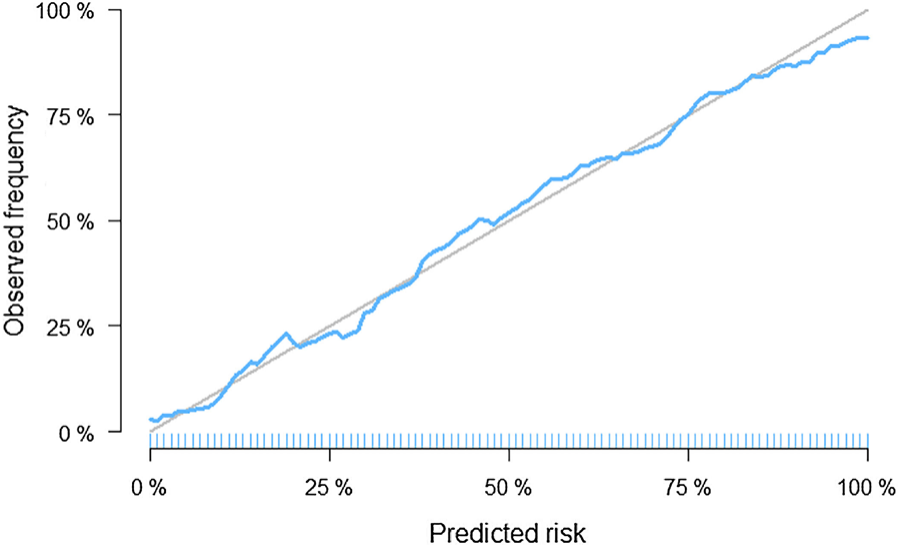
Calibration curve (blue) of the rule-based method applied on the sepsis data. The ideally calibrated one is shown in grey

The rule-based method used in our study is able to predict the prognostic outcome of the sepsis patients with the final rule set. See the calibration curves given in Fig. 4 which show that the rule-based calibration curve (blue curve) is close to the ideally calibrated curve (grey curve). We compare the rule-based model with a variety of baseline machine learning models on the performance of in-hospital death prediction, through the procedure illustrated in the prediction analysis section.

**Table 3:** Performance of rule-based model vs. baseline models

Method	Mean AUC (SD)	*P* value
SAPS-II	**0.794 (0.017)**	$<0.01$
LODS	0.757 (0.016)	$<0.01$
SOFA	0.743 (0.020)	$<0.01$
qSOFA	0.56 (0.018)	$<0.01$
SIRS	0.564 (0.018)	$<0.01$
SVM	0.675 (0.015)	$<0.01$
Random Forest	0.687 (0.017)	$<0.01$
LASSO	**0.766 (0.018)**	$<0.01$
Ridge regression	0.765 (0.017)	$<0.01$
Logistic regression	0.765 (0.018)	$<0.01$
Rule-based	**0.781 (0.018)**	-

The third column (p value) shows the significance of difference in AUCs (obtained via the Delong test) of each model against the rule-based model. The largest three AUC values are shown in bold

Apart from the above machine learning models, comparison is also made between the rule-based method and several scoring systems commonly used in clinical practice. These scoring systems are SAPS-II, LODS, SOFA, qSOFA, and the SIRS criteria [50] . The computation of these scores for each patient is accomplished with the help of severity scores SQL scripts from the mimic-code repository [51]. Note that the computation of these scores does not necessarily require exactly the same features as the rule-based method. SIRS and qSOFA use only a small fraction of features incorporated in the sepsis data. SOFA and LODS incorporate similar variables as we do while SAPS-II requires much more features which are not limited to the range of variables in our sepsis data set.

Results of the performance comparison is shown in Table 3. As can be seen, SAPS-II, rule-based method, and LASSO are among the top three methods that best predict in-hospital death events of sepsis population (shown in bold). The mean AUC of rule-based method is higher than all other models except SAPS-II (*p* value $\le 0.001$), which demonstrates that rule-based method is able to yield a comparable or even better prediction result on the sepsis population compared with most common scoring systems. Although the rule-based model is slightly inferior to SAPS-II with regard to prediction power, the rule-based method outweighs SAPS-II in terms of features incorporation and interpretability. By “interpretability” we mean it helps us understand the sepsis syndrome as well as its population via potential rules. These rules not only provide us with a handy tool for risk prediction, but may enlighten us on the complex relationship between the risk factors of sepsis which will be discussed in detail in the next section.

### External validation with the PhysioNet computing in cardiology challenge 2012 data

#### The PhysioNet/CinC challenge 2012 data

As external validation has been given high priorities in many studies recently [52, 53], we implement complementary experiments to test whether the rule-based method as well as the rules identified with the MIMIC-III data make good predictions on another similar data: The PhysioNet Computing in Cardiology Challenge (PNCCC) 2012 Data [54] which was collected and provided to its participants for the development of methods for prediction of mortality rates in ICU populations.

The PNCCC 2012 data perfectly meets our requirements for external data in this study. The exact data consists of medical records ranging from general demographics to hourly vital sign measurements of 4,000 patients from their first two days admitted to the ICU, covering all the routine risk factors we incorporate in our study. Common clinical outcomes (length of ICU stay, in-hospital death, etc) and acuity scores (SAPS-I, SOFA, etc) are also available. The criterion for subject inclusion are the same as the MIMIC-III sepsis data except that we do not use the ICD-9 for sepsis diagnosis due to the absence of this code in the PNCCC data. Instead, we define the sepsis cohort with the following criteria: (i) meet two components of SIRS criteria (ii) SOFA$\ge 2$ which, to the greatest degree, identifies the sepsis-related subjects. Since part of the patient inclusion criteria for sepsis diagnosis alters, this experiment can be viewed as a robustness check that investigates how the rule-based model performs when variation of data for model development is introduced. The final validation data set involves records of 1468 sepsis patients. The predictors incorporated are handled (in terms of feature extraction and missing value imputation) exactly the same way as we handle the MIMIC-III sepsis data set.

#### Results on the PhysioNet/CinC challenge 2012 data

To show how well the rule-based method and rules we developed on the MIMIC-III sepsis data predicts mortality on the external sepsis data, we simply check the endorsement of rules on the new samples and fit a rough logistic regression model, instead of identifying new rules with the new data. Other baseline machine learning models are fitted and common scores are evaluated, both for testing their performance on the complete records of 1468 patients from the PNCCC data.

We list the results of each model’s AUC on in-hospital mortality prediction and the *p* value of the Delong test in Table 4. Obviously, the rule-based method outperforms other baseline methods and scoring systems with an AUC of 0.788, significantly higher than that of other models (all *p* values $< 0.05$ ).Therefore, we argue that the rules identified in our study can be reasonably applied to a group of patients in a similar setting.

**Table 4:** Performance of the rule-based model vs. baseline models applied on the PNCCC 2012 data

Method	AUC	*p* value
SAPS-I	0.612	$< 0.01$
SOFA	0.584	$< 0.01$
qSOFA	0.502	$< 0.01$
SIRS	0.505	$< 0.01$
SVM	0.716	0.014
Random Forest	0.532	$< 0.01$
LASSO	0.711	$< 0.01$
Ridge regression	0.709	0.021
Logistic regression	0.707	0.017
Rule-based	0.788	–

The third column (*p* value) shows the significance of difference in AUCs (obtained via the Delong test) of each model against the rule-based model

## Discussion

We apply RuleFit on a set of routinely collected measurements with which we expect to identify a set of informative rules to determine the death risk of sepsis individuals. The factors involved in the final rule set include vital signs such as MAP (mean arterial pressure), heart rate and GCS, while others are lab values like serum potassium, serum sodium, serum creatinine, serum bilirubin, serum albumin, platelets, and arterial pH. Age, as demographic information, has also been identified as important. The existence of these variables in the final rules is able to determine the presence of situations like trauma, infection, organ dysfunction or failure, respiratory distress and many other diseases that are prevalent among sepsis patients.

**Table 5:** References for the top 10 rules

Literature	Rule									
	865	919	1780	1798	1231	1609	249	1145	777	655
Adeletti et al. [55]		$\checkmark$	$\checkmark$	$\checkmark$					$\checkmark$	
Goyal et al. [56]		$\checkmark$		$\checkmark$					$\checkmark$	
Kurowski et al. [57]					$\checkmark$	$\checkmark$		$\checkmark$	$\checkmark$	$\checkmark$
Wang [58]						$\checkmark$				$\checkmark$
Solinger and Rothman [59]		$\checkmark$		$\checkmark$					$\checkmark$	
Fischbach and Dunning [60]		$\checkmark$								
Lippi et al. [61]		$\checkmark$								
Robson et al. [62]		$\checkmark$								
Patel [63]						$\checkmark$		$\checkmark$		$\checkmark$
Zhai et al. [64]						$\checkmark$		$\checkmark$		$\checkmark$
Sedlak and Snyder [65]						$\checkmark$		$\checkmark$		$\checkmark$
Marconi et al. [66]						$\checkmark$		$\checkmark$		$\checkmark$
Temme et al. [67]						$\checkmark$				$\checkmark$
Boland et al. [68]						$\checkmark$				$\checkmark$
Heitkemper et al. [69]			$\checkmark$		$\checkmark$		$\checkmark$			
Akhter et al. [70]			$\checkmark$		$\checkmark$		$\checkmark$			
Gogos et al. [71]	$\checkmark$						$\checkmark$			
Suetrong and Walley [72]	$\checkmark$						$\checkmark$			
Dellinger et al. [73]	$\checkmark$						$\checkmark$			
Kraut and Madias [74]	$\checkmark$						$\checkmark$			
Leibovic [75]					$\checkmark$			$\checkmark$		
Baygin and Kararmaz [76]					$\checkmark$			$\checkmark$		
Pisani [77]	$\checkmark$					$\checkmark$				
Dünser [78]				$\checkmark$						

Each column lists the reference for a rule (with a tick mark)

We first give a brief description of these factors: GCS is used to evaluate the status of central nervous system, i.e., the degree of a person’s consciousness. Platelets help the body form clots to stop bleeding when the blood vessels gets damaged. Potassium, sodium, creatinine, bilirubin and albumin are all components of blood serum whose main role is to carry blood cells, transport life-sustaining substances and wastes produced in the body, and maintain the balance of blood. In detail, potassium and sodium maintain the electrolyte balance in the blood cell; creatinine is the byproduct of muscle metabolism which is carried by blood and removed by kidneys; bilirubin is one of the main metabolic wastes of the human body which is antioxidative yet toxic; and albumin helps keep fluid in the bloodstream to ensure that blood does not leak into other tissues. Levels of these components in the serum are often used to evaluate how well the main human body organs function, and significant changes in the level of these components may indicate organ dysfunction such as liver disease or kidney failure, respiratory distress and so on, which are commonly seen in patients with severe sepsis or septic shock [55].

The derived rules shown in Table 2 provide a good estimation of in-hospital mortality risk for the sepsis cohort in our study, and the factors involved in the rules have demonstrated their significance in a number of studies on the prediction of clinical outcome for ICU patients. Most of the rules are able to identify meaningful or widely accepted cut-off values for the risk factors involved (note that the cut-off values of a risk factor mentioned in our study are limited to its worst observation within 24hr of ICU admission), although some rules reveal various thresholds for a given risk factor. For this kind of rule, we recommend further investigation on the relevant risk factors as well as their interactions with each other. We then give a detailed illustration of the top 10 rules generated by RuleFit as well as the factors that constitute the rules. The interpretation of each rule is based on a wide range of relevant previous studies and in order to be slim, we list the literature each rule refers to in Table 5.

As we can see from these rules, GCS, serum potassium, and bilirubin are identified as the three factors most involved in predicting in-hospital mortality as they are the factors with the most frequent occurrence in the whole rule set. In [79], it is demonstrated that GCS is one of the strongest predictors of outcome in their multivariate model for patient risk stratification in the ICU. Besides, compared with other commonly-measured values in scoring systems like APACHE II and SAPS II, both GCS and bilirubin have shown direct correlation to the outcome in their study [19, 20]. Studies like [56, 80] also manifest that the level of serum potassium is associated with mortality of many diseases prevalent in sepsis patients, such as Acute Myocardial Infarction, heart failure, etc.

GCS is used as part of many ICU scoring systems including SAPS II, SOFA, and APACHE II. This scale has contributed a great extent of predictive power to APACHE II and has demonstrated its prognostic importance on ICU and hospital mortality rates at admission levels [57]. Generally, a GCS score above twelve is classified as mild disturbance of consciousness, a nine to twelve as moderate, and a score below nine as severe disturbance. Higher score generally associates with lower risk of mortality. The cut-off values in rule 777, 1231, and 1145 are consistent with the thresholds of GCS, as GCS greater than eight in rule 777, nine in rule 1231 and rule 1145 respectively is considered to be risk-decreasing. In rule 655 and rule 1608, the cut-off value for GCS is five and GCS is combined with different levels of bilirubin. [58] states that the level of serum bilirubin correlates with mortality in patients with traumatic brain injury. Although lower GCS and higher level of serum bilirubin were observed among non-survivors in their study, the general relationship between serum bilirubin and GCS, together with the influence of their relationship on patient mortality remains a mystery, due to the complex role bilirubin plays in the human body.

Rules 777, 919 and 1798 identify 4.2 mMol/L (or close to 4.2 mMol/L) as the threshold of serum potassium in determining higher risk versus lower risk group of patients. This may suggest a dramatic trend downward for patients with sepsis, which is to say, a safe range of serum potassium for patients with sepsis becomes 3.7-4.2 mMol/L, instead of 3.7-5.1 mMol/L for a healthy adult [55]. Some studies may provide evidence for this new range: [59] found a mortality risk below the population average when potassium is between 3.4 and 4.3 mMol/L and [56] observed the lowest mortality in Acute Myocardial Infarction (AMI) inpatients whose post-admission serum potassium level is between 3.5-4.5 mMol/L, which may also be the case for sepsis, given the complexity of the disease. Note that rule 919 might reflect an interesting finding on the relationship between the level of serum potassium and platelets demonstrated in relevant research. According to [60], hyperkalemia is defined as potassium exceeding 5.5 and is associated with significant morbidity and mortality. In [61, 62], however, platelets would release extra potassium and hence cause peudo-hyperkalemia. Rule 919 suggests that when the level of platelets is lower than 137, a potassium level higher than 4.1 may indicate a real hyperkalemia since we can not attribute extra potassium to a high level of platelets. Due to a frequent observation of hyperkalemia in patients with renal failure, it is reasonable to say that rule 919 may be able to recognize sepsis patients with peudo-hyperkalemia. The slight difference between the cut-off values of potassium, (i.e., 4.1 and 4.25) may be due to their interactions with other risk factors. However, in rule 1780, the cut-off value of potassium turns to 3.3, and we suggest further investigation of this since there is currently little research on how serum potassium and creatinine interact.

Our rules recognize three levels for serum bilirubin, i.e., 1.15 in rule 1608, 7.5 in 655, and 20 in rule 1145. These rules may indicate a patient in risk-decreasing condition in different scenarios. The reference interval of bilirubin is 0.3–1.0 mg/dL for adults, and a higher level of bilirubin may be defined as hyperbilirubinaemia which occurs frequently in neonates and is a common complication of sepsis. As a biomarker of liver function, bilirubin is toxic and can make irreversible damage to the brain and neural system. Several studies have shown that elevated serum bilirubin levels may induce inflammation, apoptosis, and sepsis-related acute respiratory distress syndrome (ARDS) [63, 64] and in particular for patients with liver disease, extra bilirubin leads to worse clinical outcomes. Nevertheless, a high bilirubin level also confers various health benefits [65, 66], instead of high risk, especially for patients without liver disease. For example, bilirubin has been shown to be protective against cardiovascular disease (CVD) and high serum bilirubin within normal ranges was associated with low cancer mortality in a Belgian population due to the antioxidant activity of bilirubin [67]. Thus, it is reasonable to consider the specific patient condition when determining the effect of bilirubin on clinical outcomes.

In our case, the threshold of 20 in rule 1145 might indicate that a high level of bilirubin might not do considerable damage to the brain and in contrast, might bring much benefit to the heart and decrease the risk of death. Rule 1608 identifies a bilirubin level $\le$ 1.15 as risk-decreasing, provided that age $\le$ 81 and GCS >5. In rule 655, the age constraint no longer exists and the cut-off value ascends to 7.5. These two rules as a whole indicate the interaction between age and bilirubin shown in [68], illustrating that bilirubin levels gradually increase with age in older adults and elevated bilirubin in older individuals is not associated with improved survival.

The most commonly used indicator of renal function is serum creatinine, the reference interval of which is 0.4–1.3 mg/dL [69]. According to [70], an increase in serum creatinine by 0.5mg/dL has an independent adverse influence on clinical outcomes such as length of stay, rate of readmission as well as six-month mortality. Rules 1780 and 249 find that, in accordance with the reference interval, serum creatinine $\le$ 1.2 mg/dL indicates risk-decreasing and creatinine > 1.2 indicates risk-increasing. The interaction between creatinine, potassium, and arterial pH is apparently natural since they both reflect the degree of blood cell balance in human body. Rule 1231 determines a patient in high or low risk by assessing how well the heart, kidney and brain function. The 1.7 for creatinine in rule 1231 is a high threshold with little supporting evidence at present and more investigations are expected.

Arterial pH is also recognized as an important risk predictor in our rules. This is consistent with existing experience of [71]. When pH descends to a level below the normal range, i.e., 7.35–7.45, it may imply the potential presence of acidosis that leads to unfavorable outcomes in ICU patients. For example, [81] indicates that the admission value of arterial pH contributes to the severity of traumatized patients. [72] demonstrates that admission pH of lactic acidosis patients is associated with significant morbidity and mortality. While the Survival Sepsis Campaign (SSC) recommends treatment of acute metabolic acidosis if pH < 7.1 in severe sepsis and septic shock patients [73], our rules identify the arterial pH threshold distinguishing the higher risk from the lower risk at 7.2. As the study of [74] suggests that metabolic acidosis might be beneficial for oxygen delivery and metabolism, a slight upward adjustment of pH cut-off value may not always be harmful. Besides, there may be interactions between pH, creatinine, and age as rule 249 and 865 indicate, which needs to be further explored in the future.

In rule 1231 and rule 1145, heart rate below 129 bpm and 133 bpm respectively is considered to be risk-decreasing. Even though these two levels of heart rate are far beyond the normal resting heart rate of 60–100, this is not unusual as sepsis has strong relationship with tachycardia, defined as heart rate that exceeds the normal resting rate. It has been shown that because of excessive inflammation and circulating stress hormones, sepsis patients often experience tachycardia, the duration of which may be related with mortality [75, 76]. Rules 1231 and 1145 may indicate that under mild unconsciousness (GCS $\ge$ 9), the presence of slight tachycardia may not always put patients in fatal danger, which might partly associate with the cardiovascular benefits from a high level of bilirubin, or somehow relate to an acceptable high level of creatinine.

In addition to the above thresholds, the derived rules also identify meaningful or potential risk thresholds for other predictors: rule 777 identifies albumin > 1.9 g/dL as risk-decreasing. In fact, the cut-off of 1.9 is much lower than the normal lower bound of serum albumin, i.e., 3.4g/dL. Though low albumin levels may be induced by liver or kidney disease, it is still not clear how low the level of serum albumin increases significantly the death risk of sepsis patients. Older age has been recognized as one of the most powerful clinical prognostic indices of death in many studies like [71, 77]. Specifically, age > 65 (the general definition for being elderly) has been proven as an independent risk factor in mortality prediction. As to our population, rule 865 and rule 1608 suggest that more refined age classification is needed for estimating sepsis mortality. For MAP, rule 1798 identifies the cut-off value to be 59 mmHg, which is close to the value in the conclusion of [78] showing that MAP $\ge$ 60 mmHg may be as safe as higher levels during the first 24 h of ICU therapy in septic patients.

## Conclusion

This study explores sepsis data from the MIMIC-III database by utilizing a rule-based method which produces a refined rule ensemble for the purpose of in-hospital death prediction. The application of rule-based method in our study yields comparable prediction performance compared to many baseline medical scoring systems and machine learning classifiers and has an ability to improve our understanding of different risk patterns of sepsis population through the derived risk-predictive rules. The top 10 rules identified by the method have found that GCS, serum potassium and serum bilirubin are among the most important risk factors for mortality prediction of the population in our study, and the interactions between these risk factors may change or influence what we have known about the effect of a risk factor on the patient outcome.

Our work may contribute to the community with regard to the following aspects: Firstly, our work highlights that existing studies, especially machine-learning-based research, fail to discuss how much insight and understanding their models could provide to the study of sepsis and its population. Hence, we discuss in detail the rules identified for the sepsis cohort. The cut-off values of risk factors and the interactions between these risk factors may suggest underlying disease patterns of sepsis patients. All of these implications may enlighten research on potential relationship between certain risk factors indicated in the rules, and thereby eventually improve our understanding of sepsis prognosis. Secondly, despite the need for further validation, the rules found in our research may be of value in fast risk prediction in real clinical practice due to the implications hidden in the rules and the convenient form of rules. Thirdly, our study demonstrate the power of RuleFit to find clinically informative rules. RuleFit could enrich the toolkit of sepsis prognosis and many other common conditions in the ICU. Finally, it is worth mentioning that our work provides a good example of predicting sepsis mortality with only a small number of variables, a challenge mentioned in [71] as the data used in our work only require routinely collected and easily acquired vital signs, a few lab tests and some demographic information of sepsis patients. Thus, our method could be easily applied on a regular basis.

There are limitations in our application of rule-based method with regard to the RuleFit method and the data we use. On the one hand, RuleFit is unable to find all but a subset of possible informative rules and interactions of the variables given to the model, thus similar to other models, RuleFit should be applied in a complementary way together with other tools. Apart from this, RuleFit may yield too many rules when applied to high-dimensional data that cannot be understood quickly by the human experts, even with the integrated method of LASSO to enhance sparsity. Our study simply reports the 10 top rules of the 77 rules identified by RuleFit. A large number of rules may require much efforts in interpretation. On the other hand, the ICD-9 code, used in our study to identify sepsis patients, is designed primarily for billing purposes, hence we could not ensure the subjects in our study are all accurately diagnosed with sepsis. Besides, the data we extracted from MIMIC-III suffers from a large quantity of missing data which could be partially attributed to the difficulty in determination of precise and well-acknowledged item ids for each variable. Due to the above reasons, we expect extensive validation of the rules identified in our study on other sepsis populations.

Improvements could also be made to the application of RuleFit for sepsis. So far, our application only yields comparable prediction performance in comparison with the baseline scoring models and machine learning classifiers. More risk factors could be included since recently there exist several biomarkers which have proven to be predictive in sepsis prognosis [82, 83]. In addition, we only consider the worst value of each variable within the first day of ICU admission. Future work may incorporate longitudinal data that reflects the change of risk factors and progression of patient condition to make better predictions. Notably, before the rules are validated and used in reality, it is also necessary to set up a filtering mechanism when RuleFit produces a large number of rules, which calls for expertise in both sepsis and the RuleFit method.

## Supplementary information

**Additional file 1.** The remaining 67 rules.

**Additional file 2.** TRIPOD Checklist for prediction model development and validation with added text excerpts or remarks.

**Additional file 3.** Rule filtering criteria and the definition of the worst value within 24hrs of ICU admission.

## Data Availability

The data set generated and/or analysed for model development during the current study are not publicly available as it is based on the raw data from MIMIC-III whose acquisition involves a required training and corresponding credentials. The mimic-iii data is available in the MIMIC-III repository, https://mimic.physionet.org/gettingstarted/access/ The MIMIC Code Repository contains collaboratively developed code for a large number of useful concepts in MIMIC, see https://github.com/MIT-LCP/mimic-code The PNCCC 2012 data used for external validation provides open access to any users, available from https://www.physionet.org/content/challenge-2012/1.0.0/

## Abbreviations

ICU: Intensive care unit
AUC: Area under the curve
GCS: Glasgow Coma Scale
MIMIC: Medical Information Mart for Intensive Care
MAP: Mean arterial pressure
pH.art: Arterial pH
KM curves: Kaplan-Meier curves
APACHE: Acute Physiology And Chronic Health Evaluation
SAPS: Simplified Acute Physiology Score
SOFA: Sepsis-related Organ Failure Assessment
MODS: Multiple Organ Dysfunction Score
LODS: Logistic Organ Dysfunction Score
SIRS: Systemic Inflammatory Response Syndrome
qSOFA: quick Sequential (Sepsis-related) Organ Failure Assessment score
PNCCC: The PhysioNet Computing in Cardiology Challenge 2012
