# Supplementary material for: Understanding the complexity of sepsis mortality prediction via rule discovery and analysis: a pilot study
Source: BMC Med Inform Decis Mak. 2021 Nov 28;21:334. doi: 10.1186/s12911-021-01690-9 (PMC8628441; doi:10.1186/s12911-021-01690-9)
Supplement: Supplementary file 1 — Additional file 1. The remaining 67 rules. [file 12911_2021_1690_MOESM1_ESM.docx]

**Appendix**

**A. Rule filtering criteria based on the rule analysis Procedure.**

1. Specify a p-value threshold p_0_ for all rules, here we used p_0_=0.001.
2. For each rule in the final rule set, retain the rules that satisfy the following two requirements and discard those that do not:
   1. All p-values of the revised rules are greater than p_comp_ and less than p_0_.
   2. The p-value of the complete rule (p_comp_) is less than p_0_.
3. For each rule in the final rule set, retain the rules that satisfy the following two requirements and discard those that do not:
   1. The p-value of the complete rule (p_comp_) is less than p_0_.
   2. All p-values of the revised rules are greater than p_0_.
4. Combine the retained rules and obtain the final rule set.

**B. The definition of the worst value within 24hrs of ICU admission.**

To illustrate the definition of the worst value within 24hrs of a specific risk factor, we first introduce several reasonable ranges of this factor. This factor is associated with thresholds to detect outliers as well as more refined thresholds to define a physiologically valid and normal range of measurements.


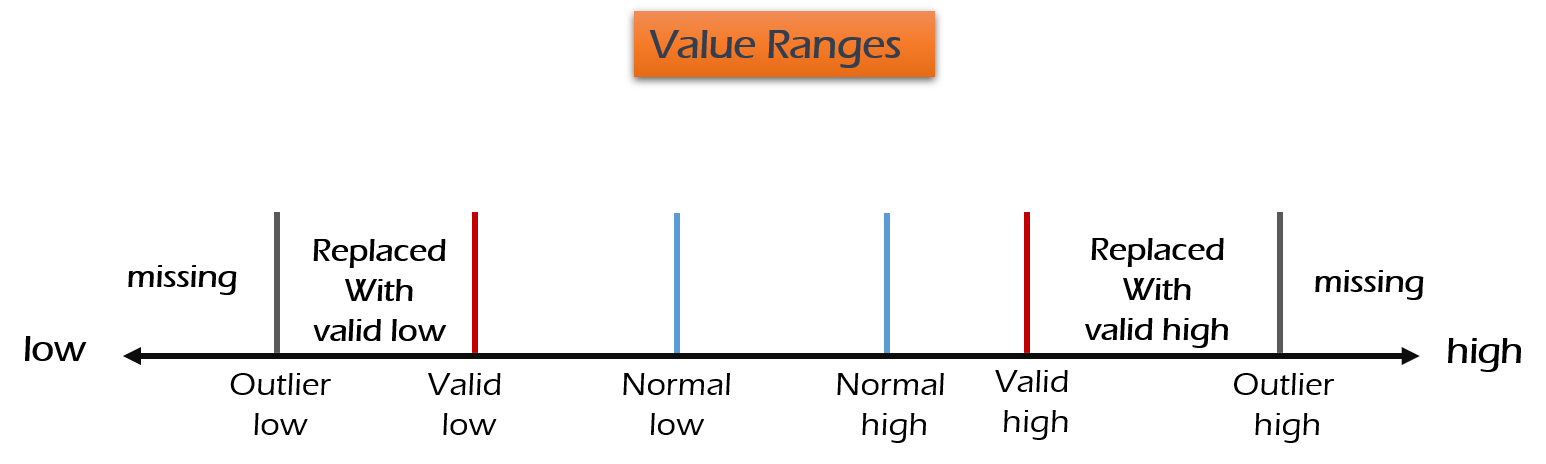


**Figure 1. Worst value definition.** Value line of a given risk factor. For a given risk factor, multiple ranges are defined. Interval within the blue vertical bars is the normal range for a given risk factor, red bars valid, and gray non-outlier.

As illustrated in Figure 1, on the one-dimension value line, several intervals are defined. From the outlier low (left gray vertical line) to outlier high (right gray vertical line) is the non-outlier interval of this risk factor. From the valid low (left red vertical line) to valid high (right red vertical line) is the valid range of this risk factor. From the normal low (left blue vertical line) to normal high (right blue vertical line) is the normal range of this risk factor. Any non-outlier value that falls outside the physiologically valid range is replaced with the nearest valid value. We then calibrate these invalid values or outliers: any value that falls outside the non-outlier area would be treated as a missing value and imputed by a common shared value. The list of these ranges for common lab values and physiological measurements can be found in <https://github.com/MLforHealth/MIMIC_Extract> .

Here, we take MAP for an example: The non-outlier range, physiologically valid range, and normal range of MAP are 0-375 bpm, 14-330 bpm, and 60-100 bpm respectively. 0 is the threshold for a low non-outlier and 375 is the threshold for a high non-outlier. Similarly, we have 14 for a valid lowest MAP, 330 for a valid highest MAP, 60 for a normal lowest MAP, and 100 for a normal highest MAP. According to this range, any MAP value that is lower than 14 (higher than 330) would be considered as an invalid value and then replaced by 14 (330). Any value that is lower than 0 (higher than 375) would be considered as an outlier and treated as a missing value. We impute 77 for the missing MAPs.

With the above ranges defined, we are able to define the worst value within 24hrs of ICU admission of a given risk factor as the most deviated value from the normal range of this risk factor. Specifically, we first compute the deviated amount of each value to its nearest valid threshold if the value falls inside the valid range and outside the normal range. If the value falls inside the normal range, then the deviated value is 0, otherwise it is greater than 0. The worst value is the exact value with the greatest deviated amount and if all values obtained within 24hrs are within the normal range, that is, all deviated values are 0, the worst value is defined as the mean value.
