# Supplementary material for: Understanding the complexity of sepsis mortality prediction via rule discovery and analysis: a pilot study
Source: BMC Med Inform Decis Mak. 2021 Nov 28;21:334. doi: 10.1186/s12911-021-01690-9 (PMC8628441; doi:10.1186/s12911-021-01690-9)
Supplement: Supplementary file 2 — Additional file 2. TRIPOD Checklist for prediction model development and validation with added text excerpts or remarks. [file 12911_2021_1690_MOESM2_ESM.pdf]

Table 1. The remaining 52 rules used to predict in-hospital death events for the sepsis patients.

number	description	direction	support	p-value	p-value：1st_removed	p-value：2nd_removed	p-value：3rd_removed
1602	creatine > 1.2 & sbp <= 99.8 & mean.bp <= 58	increasing	10%	0	0	0	0
1742	FiO2 <= 0.8 & bilirubin <= 1.15 & age <= 65.7	decreasing	20%	0	0	0	6.90E-10
1103	ph.art > 7.1 & sodium > 130 & FiO2 <= 0.8	decreasing	76%	0	0	0	0
1052	ph.art > 7.1 & temperature > 35.4 & bilirubin <= 7.3	decreasing	71%	0	0	0	0
952	potassium.serum <= 4.1 & albumin > 1.5 & gcs > 5.1	decreasing	56%	2.22E-16	1.24E-07	6.66E-16	1.74E-12
1890	FiO2 <= 0.8 & age <= 60.8 & hematocrit > 24.5	decreasing	25%	0	7.77E-16	2.33E-14	0
1765	potassium.serum > 3.9 & sbp <= 104.8 & age > 40.7	increasing	21%	0	0	6.01E-13	0
1644	FiO2 <= 0.75 & bilirubin <= 1.05 & age <= 76.7	decreasing	27%	0	1.19E-14	0	8.85E-10
1737	FiO2 <= 0.75 & sodium > 126	decreasing	79%	0	4.85E-10	0	
1730	ph.art > 7.25 & age <= 77.7 & ph.art <= 7.45	decreasing	38%	0	5.48E-08	7.82E-14	0
859	potassium.serum <= 4.3 & FiO2 <= 0.65 & heart.rate <= 114	decreasing	30%	6.97E-08	0.000846	5.89E-06	2.88E-10
597	ph.art > 7.2 & bilirubin <= 12.1 & heart.rate <= 92.4	decreasing	33%	5.81E-06	0.345054	4.22E-05	7.77E-16

number	description	direction	support	p-value	p-value：1st_removd	p-value：2nd_removed	p-value：3rd_removed
581	gcs > 7.45 & potassium.serum <= 5.3 & heart.rate <= 114	decreasing	44%	7.56E-07	0.0066	0.000116	1.69E-12
1224	gcs > 5.2 & age <= 58.2 & bilirubin <= 5.9	decreasing	24%	0	0	9.64E-14	2.68E-14
546	gcs > 9.2 & age <= 73.5 & bilirubin <= 2.85	decreasing	25%	6.66E-16	1.11E-16	3.00E-09	3.08E-12
1506	ph.art > 7.05 & potassium.serum <= 4.8 & FiO2 <= 0.85	decreasing	75%	0	0	0	0
1252	gcs > 5.1 & bilirubin <= 7.2	decreasing	88%	7.62E-14	2.21E-07	3.61E-07	
1640	gcs > 6.2 & age <= 59.5 & bilirubin <= 2.85	decreasing	21%	0	0	1.23E-11	5.22E-15
341	gcs > 5.2 & potassium.serum <= 4.05 & temperature > 35.4	decreasing	44%	1.11E-16	1.38E-13	1.11E-16	1.89E-15
634	gcs > 11 & bilirubin <= 5.3 & albumin > 1.9	decreasing	30%	2.83E-05	1.29E-07	0.000539	0.000117
1343	gcs > 7.5 & temperature <= 35.4 & platelets <= 144	increasing	5%	3.22E-07	1.40E-11	0.147763	2.22E-06
1612	gcs > 5.1 & bilirubin > 1.15 & FiO2 > 0.65	increasing	12%	5.42E-08	1.17E-10	0.008439	0.012696
1536	temperature > 35.2 & age <= 61.5 & bilirubin <= 4.6	decreasing	27%	0	0	3.89E-13	1.11E-16
642	ph.art > 7.05 & bilirubin <= 6.6	decreasing	89%	0	9.18E-08	0	
498	ph.art <= 7.2 & bilirubin > 2.3	increasing	9%	7.91E-13	0.000158	3.29E-14	
453	potassium.serum <= 4.8 & temperature > 34.5 & FiO2 <= 0.8	decreasing	73%	0	0	0	1.04E-14
1887	potassium.serum <= 5.05 & albumin > 2	decreasing	80%	1.82E-10	0.004967	4.28E-12	
347	ph.art > 7.2 & creatine <= 2.1	decreasing	55%	4.57E-14	1.65E-05	3.29E-14	
1510	gcs > 6.4 & temperature <= 35.6 & sodium <= 133	increasing	6%	1.65E-10	4.76E-13	0.159862	6.33E-08

number	description	direction	support	p-value	p-value：1st_removd	p-value：2nd_removed	p-value：3rd_removed
1807	gcs <= 12.7 & bilirubin <= 6.6 & age > 63.9	increasing	45%	1.97E-08	1.77E-08	4.81E-10	0.150203
971	gcs > 7.5 & potassium.serum <= 4.7 & heart.rate > 87.9	increasing	39%	0.000952	0.016123	0.003704	3.35E-12
1578	ph.art <= 7.2 & age > 51.3	increasing	24%	0	2.23E-09	3.29E-14	
1278	ph.art > 7.2 & gcs > 11.35 & sodium > 130	decreasing	25%	0.000242	0.002511	1.11E-16	0.000968
1163	gcs > 5.2 & temperature > 35.6 & bilirubin <= 6.7	decreasing	65%	0	0	1.10E-14	0
464	ph.art > 7.2 & potassium.serum <= 4 & sbp <= 110.9	decreasing	16%	0.00018	0.018068	0.5018	4.91E-14
1897	age <= 61.5 & bilirubin <= 5.2 & ph.art > 7.05	decreasing	29%	0	0	0	0
246	ph.art <= 7.2 & age > 45.7	increasing	27%	0	1.74E-09	3.29E-14	
1152	temperature > 35.6 & gcs > 7	decreasing	62%	0	5.05E-08	5.44E-15	
18	gcs > 9.7 & bilirubin <= 7.5 & creatine <= 1.7	decreasing	34%	2.38E-10	2.13E-11	1.13E-08	1.64E-07
1831	FiO2 <= 0.75 & heart.rate <= 133 & potassium.serum <= 5.05	decreasing	63%	0	1.09E-07	0	2.61E-13
1487	gcs > 7.5 & temperature > 35.4 & age <= 80.2	decreasing	50%	0	0	2.22E-16	0
1622	age > 57 & sodium <= 135.7	increasing	18%	3.07E-06	0.005842	5.17E-13	
198	gcs <= 9.2 & FiO2 > 0.75 & albumin <= 2.8	increasing	8%	0	0	4.30E-07	0
1691	bilirubin <= 6.65 & age <= 58.1	decreasing	26%	0	1.64E-12	9.71E-08	
1696	bilirubin <= 7.6 & age <= 64 & bilirubin <= 1.4	decreasing	23%	0	0	0.001003	1.11E-16

number	description	direction	support	p-value	p-value：1st_removd	p-value：2nd_removed	p-value：3rd_removed
1669	potassium.serum <= 4.85 & temperature > 34.5 & gcs > 4.2	decreasing	79%	0	5.74E-11	3.33E-16	5.55E-16
1514	bilirubin <= 7.15 & age <= 47 & potassium.serum <= 5.45	decreasing	11%	9.36E-13	6.80E-10	8.06E-13	2.68E-11
1581	potassium.serum <= 4 & gcs > 4.4 & mean.bp <= 264	decreasing	50%	3.00E-15	6.97E-06	7.76E-12	1.75E-14
671	gcs > 5.2 & potassium.serum <= 4.25 & bilirubin <= 7.3	decreasing	59%	0	7.49E-14	1.89E-14	3.33E-16
8	gcs > 8.35 & creatine <= 1.7 & ph.art > 7.2	decreasing	40%	3.65E-11	2.22E-15	1.55E-08	2.47E-10
553	gcs > 7.5 & temperature > 36.8 & bilirubin <= 6.65	decreasing	35%	0	0	6.06E-12	0
1131	gcs <= 9.65 & FiO2 > 0.65	increasing	19%	5.50E-14	1.16E-06	6.51E-06	

Table 2. The 15 discarded rules

number	description	direction	support	p-value	p-value：1st_removed	p-value: 2nd_removed	p-value：3rd_removed
948	potassium.serum > 4.1 and sbp > 94.1 and mean.bp > 42	decreasing	27%	0.34452	0	0.361816	0.033909
1830	FiO2 <= 0.75 and heart.rate <= 133	decreasing	69%	2.61E-13	0.022875	0	
1415	albumin > 2.4 and ph.art > 7.1 and bilirubin <= 12.4	decreasing	60%	1.37E-11	0	0.000486	1.27E-09
1731	ph.art > 7.25 and age <= 77.7 and ph.art > 7.45	increasing	5%	0.240173	0.240173	0.441722	0
287	ph.art <= 7.2 and FiO2 <= 0.85 and bilirubin <= 2.3	decreasing	18%	0.753724	7.82E-12	0.001164	0.016648
896	gcs > 9.05 and heart.rate <= 65 and FiO2 <= 0.75	decreasing	12%	0.002457	0.136516	3.34E-08	0.01719
774	potassium.serum > 4.2 and mean.bp > 50 and bilirubin <= 7.4	decreasing	18%	0.026338	4.44E-16	4.22E-06	0.278865
900	gcs > 9.05 and heart.rate > 65 and age > 56.9	increasing	29%	0.3459	1.62E-07	0.800282	7.02E-05
1480	bilirubin <= 7.6 and age <= 59.7 and bilirubin <= 1.2	decreasing	17%	5.55E-16	5.55E-16	0.000574	0
561	gcs > 9.05 and temperature > 36.5 and age <= 73.5	decreasing	22%	1.44E-15	0	4.31E-12	1.72E-11
309	ph.art <= 7.25 and gcs > 7.4 and temperature <= 36.5	increasing	11%	2.02E-08	0.000826	0	0.036052

696	ph.art <= 7.2 and ph.art > 7.1 and ph.art > 7.15	increasing	17%	0.744301	0	0.744301	0.700267
number	description	direction	support	p-value	p-value：1st_removd	p-value：2nd_removed	p-value：3rd_removed
1624	age > 57 and sodium <= 135.7 and hematocrit > 34.2	increasing	4%	0.003565	0.081344	0.000844	3.07E-06
843	ph.art <= 7.25 and temperature <= 36.5 and sodium <= 135.5	increasing	7%	2.59E-13	4.95E-10	1.34E-07	0
1115	gcs > 9.5 and bilirubin <= 6.7 and temperature > 36.7	Decreasing	27%	4.13E-12	0	3.82E-11	8.43E-09
