# Supplementary material for: Understanding the complexity of sepsis mortality prediction via rule discovery and analysis: a pilot study
Source: BMC Med Inform Decis Mak. 2021 Nov 28;21:334. doi: 10.1186/s12911-021-01690-9 (PMC8628441; doi:10.1186/s12911-021-01690-9)
Supplement: Supplementary file 3 — Additional file 3. Rule filtering criteria and the definition of the worst value within 24hrs of ICU admission. [file 12911_2021_1690_MOESM3_ESM.pdf]

TRIPOD Checklist: Prediction Model Development and Validation

Section/Topic	Item		Checklist Item	Page	Text Excerpt/Remark If Not Applicable
**Title and abstract**					
Title	1	D;V	Identify the study as developing and/or validating a multivariable prediction model, the target population, and the outcome to be predicted.	NA	The current title is finally adopted to show the biggest merit of the stud, i.e., interpretability. Actually, this study is an development and external validation of in-hospital mortality model for ICU sepsis patients.
Abstract	2	D;V	Provide a summary of objectives, study design, setting, participants, sample size, predictors, outcome, statistical analysis, results, and conclusions.	1	OBJECTIVES, STUDY DESIGN, SETTING, PARTICIPANTS, SAMPLE SIZE, OUTCOME, STATISTICAL ANALYSIS: "In this paper, a rule discovery […] more interpretable than most comparable models."<br>PREDICTORS: Not in abstract due to large number of predictors.<br>RESULTS: "In our experiment […] risk factors for predicting patient death."<br>CONCLUSIONS: "Our study demonstrates that […] as well as its population."
**Introduction**					
Background and objectives	3a	D;V	Explain the medical context (including whether diagnostic or prognostic) and rationale for developing or validating the multivariable prediction model, including references to existing models.	1/2	In *Introduction* subsections: "Sepsis is defined as […] remains a challenge due to the complexity and heterogeneity of sepsis."
	3b	D;V	Specify the objectives, including whether the study describes the development or validation of the model or both.	2	"In this study, we apply a rule-based method […] in helping us understand the complexity of sepsis and its population."
**Methods**					
Source of data	4a	D;V	Describe the study design or source of data (e.g., randomized trial, cohort, or registry data), separately for the development and validation data sets, if applicable.	2	DEVELOPMENT: In *Data* subsection: "We use data from the Medical Information Mart for Intensive Care database […] and 58,976 admissions in the database."<br>EXTERNAL VALIDATION: "The PhysioNet Computing in Cardiology Challenge (PNCCC) 2012 Data […] for prediction of mortality rates in Intensive Care Unit (ICU) populations."
	4b	D;V	Specify the key study dates, including start of accrual; end of accrual; and, if applicable, end of follow-up.	2	DEVELOPMENT: In *Data* subsection: "This database contains information related to patients admitted to ICUs at a large tertiary care hospital between 2001 and 2012 in the U.S."<br>EXTERNAL VALIDATION: NA. we do not provide details but refer to specific literature for more information.
Participants	5a	D;V	Specify key elements of the study setting (e.g., primary care, secondary care, general population) including number and location of centres.	2	
	5b	D;V	Describe eligibility criteria for participants.	3/9	DEVELOPMENT: "The patient inclusion criteria of our cohort are: […] for septic shock."<br>EXTERNAL VALIDATION: Not in method section, but in page 9 "we define patients with sepsis in the PNCCC data with the following criteria […] Other criterion for subject inclusion are the same for both data sets."
	5c	D;V	Give details of treatments received, if relevant.	NA	As we begin the prediction of in-hospital mortality from 24hrs of ICU admission, we do not consider treatment factors.
Outcome	6a	D;V	Clearly define the outcome that is predicted by the prediction model, including how and when assessed.	3	"The outcome in our study is defined to be […] to hospital discharge."
	6b	D;V	Report any actions to blind assessment of the outcome to be predicted.	NA	This research is a retrospective study based on public EHR data and the outcome is already blindly assessed.
Predictors	7a	D;V	Clearly define all predictors used in developing or validating the multivariable prediction model, including how and when they were measured.	3	"The worst value of each predictor within 24hr […] and used to fit the RuleFit model."
	7b	D;V	Report any actions to blind assessment of predictors for the outcome and other predictors.	NA	Predictors are determined by reference to existing literature on common scoring systems and other relevant machine learning models and are

					limited to routine records.
Sample size	8	D;V	Explain how the study size was arrived at.	NA	
Missing data	9	D;V	Describe how missing data were handled (e.g., complete-case analysis, single imputation, multiple imputation) with details of any imputation method.	3/9	DEVELOPMENT: "Missing values are imputed with the k-nearest […] (the number of nearest neighbours is set to be 5)."<br>EXTERNAL VALIDATION: Not in method section, but in page 9 "The predictors incorporated are handled […] the same way as the MIMIC-III sepsis data set."
Statistical analysis methods	10a	D	Describe how predictors were handled in the analyses.	3	"The worst value of each predictor within 24hr of ICU admission […] is computed and used to fit the RuleFit model"
	10b	D	Specify type of model, all model-building procedures (including any predictor selection), and method for internal validation.	3	TYPE OF MODEL, MODEL-BUILDING PROCEDURES: In *Overview of the rule-based method:* "We use a rule-based method to […] we give a detailed description of our work flow."<br>PREDICTOR SELECTION: "Note that the predictors involved […] source of infection can be included if available."<br>METHOD FOR INTERNAL VALIDATION: "We split the data randomly into a 70% training set and 30% test set […] and compare the corresponding AUCs."
	10c	V	For validation, describe how the predictions were calculated.	9	"The predictors incorporated are handled […] the same way as the MIMIC-III sepsis data set."
	10d	D;V	Specify all measures used to assess model performance and, if relevant, to compare multiple models.	6/7	"Each model is trained with […] while performance metric like AUC is calculated merely on the test set."<br>"we could also examine the significance of difference in AUC between [..] with the Delong test"<br>"comparison is also made between […] several scoring systems commonly used in clinical practice."
	10e	V	Describe any model updating (e.g., recalibration) arising from the validation, if done.	NA	External validation shows good performance.
Risk groups	11	D;V	Provide details on how risk groups were created, if done.	5/6	"As each rule in the rule set discovered […] in distinguishing the higher risk group from the lower risk group."<br>"we can use rules that show significant prediction power in both […] for sepsis patients."
Development vs. validation	12	V	For validation, identify any differences from the development data in setting, eligibility criteria, outcome, and predictors.	9	"Unlike using the ICD-9 code for […] are the same for both data sets."
**Results**					
Participants	13a	D;V	Describe the flow of participants through the study, including the number of participants with and without the outcome and, if applicable, a summary of the follow-up time. A diagram may be helpful.	NA	
	13b	D;V	Describe the characteristics of the participants (basic demographics, clinical features, available predictors), including the number of participants with missing data for predictors and outcome.	3	DEVELOPMENT: See Table 1 (without information on missing values).<br>EXTERNAL VALIDATION: NA.
	13c	V	For validation, show a comparison with the development data of the distribution of important variables (demographics, predictors and outcome).	NA	
Model development	14a	D	Specify the number of participants and outcome events in each analysis.	NA	
	14b	D	If done, report the unadjusted association between each candidate predictor and outcome.	6/7/8	Details are in the *Survival analysis and Decomposition analysis.*<br>Results can be found in Table 2 and Figure 3.
Model	15a	D	Present the full prediction model to allow predictions for individuals (i.e., all	6	In *Predction analysis.* Details are not given because they are deemed

specification			regression coefficients, and model intercept or baseline survival at a given time point).		trivial in our research framework.
	15b	D	Explain how to the use the prediction model.	6	"For clarification, the logistic regression model […] to be used for this end."
Model performance	16	D;V	Report performance measures (with CIs) for the prediction model.	7-9	See Table 2 for each rule and Table 3 and 4 for overall prediction with a rule set, all without CIs.
Model-updating	17	V	If done, report the results from any model updating (i.e., model specification, model performance).	NA	
**Discussion**					
Limitations	18	D;V	Discuss any limitations of the study (such as nonrepresentative sample, few events per predictor, missing data).	12	"There are limitations in our application of rule-based method with regard to […] we expect extensive validation of the rules identified in our study on other sepsis populations."
Interpretation	19a	V	For validation, discuss the results with reference to performance in the development data, and any other validation data.	9	"Obviously, the rule-based method outperforms […] can be reasonably applied to a group of patients in a similar setting."
	19b	D;V	Give an overall interpretation of the results, considering objectives, limitations, results from similar studies, and other relevant evidence.	9-12	See *Discussion* section<br>"Most of the rules are able to […] the factors that constitute the rules."
Implications	20	D;V	Discuss the potential clinical use of the model and implications for future research.	12/13	"Secondly […] the rules found in our research may be of value in fast risk prediction in real clinical practice due to […]."<br>"Improvements could also be made to […] which calls for expertise in both sepsis and the RuleFit method."
**Other information**					
Supplementary information	21	D;V	Provide information about the availability of supplementary resources, such as study protocol, Web calculator, and data sets.	14	See *Availability of data and materials* section.
Funding	22	D;V	Give the source of funding and the role of the funders for the present study.	NA	

*Items relevant only to the development of a prediction model are denoted by D, items relating solely to a validation of a prediction model are denoted by V, and items relating to both are denoted D;V.

*Some items are not applicable (NA) and thus remarks are given instead in the current study.
